# Editorial: The role of experience in children's language development: A cultural perspective

**DOI:** 10.3389/fpsyg.2022.964602

**Published:** 2022-08-18

**Authors:** Douglas Sperry, Priya Shimpi, Eliana Colunga, Lulu Song, He Sun

**Affiliations:** ^1^Social and Behavioral Sciences, Saint Mary-of-the-Woods College, Saint Mary of the Woods, IN, United States; ^2^Early Childhood Education, Mills College, Oakland, CA, United States; ^3^Psychology and Neuroscience, University of Colorado Boulder, Boulder, CO, United States; ^4^Early Childhood Education/Art Education, Brooklyn College City University of New York (CUNY), Brooklyn, NY, United States; ^5^National Institute of Education, Nanyang Technological University, Singapore, Singapore

**Keywords:** children's socio-cultural experiences, linguistic pathways, bilingualism, literacy, communication, influences on language development, children's language development, cultural perspective

This Research Topic charted a challenging course to re-envision conventional methods and assumptions in language development, a course even more current in this time of global challenge to WEIRD ways (*W*estern, *e*ducated, *i*ndustrialized, *r*ich, and *d*emocratic) and rapprochement to diverse funds of knowledge. The Research Topic begins with the assumption that attending to the diversity of social and cultural contexts in which children learn language(s) allows us to understand better how context helps shape learners' attentional and behavioral patterns.

The articles included in this Research Topic have more than risen to this occasion. A critical goal for this Research Topic was to incorporate perspectives gleaned from underrepresented voices to increase our understanding of language acquisition and enhance future endeavors to ameliorate the effects of poverty, systemic racism, and global unrest from a position that is culturally informed by the strengths that all groups bring to the table. The articles examine understudied populations as defined by cultural and socioeconomic differences (Huang and Kan; Luo et al.; Moreno et al.; Rumper et al.; Southwood et al.; Sperry and Sperry; Ward); mobility and its effects (Huang and Kan; Ward); language survival (Ward); and geographic dispersion (Moreno et al.; Sperry and Sperry; Ward). They address issues of cultural isolation (Moreno et al.; Sperry and Sperry; Ward) and cultural contact (Southwood et al.; Huang and Kan; Ward). They also help us appreciate the experiences of bilingual/bicultural individuals, either in their dealings in a new culture and land (Huang and Kan) or as long-term residents of established bilingual/bicultural communities (Luo et al.; Rumper et al.). Moreover, the researchers themselves represent diverse perspectives and positionalities. These articles and their authors challenge us to consider how very young language learners might use the panoply of social information gleaned from their heterogeneous families and communities as they sort out the mysteries of semantic, syntactic, and pragmatic features of their language.

Our second desire for this Research Topic was that it reflect the manifold approaches to the study of language acquisition. This desire was met with articles that employ traditional, Bayesian linguistic modeling (Tripp et al.); mixed methods using parental- and teacher-report questionnaires or well-established prompts to complement qualitative analyses (Huang and Kan; Luo et al.; Ward); community viewpoints in the style of participatory research (Rumper et al.); microanalysis of ethnographic investigations (Sperry and Sperry; Ward) and of moment-by-moment parent-infant social interactions (Sun et al.); quantitative analysis in service of intervention design (Moreno et al.; Rumper et al.); correlational research re-interpreting standardized assessment tools (Southwood et al.); and theoretical explorations of what language assessment and evaluation within a culturally sensitive framework should accomplish in order to oppose the current and future heads of the linguistic deprivation hydra that challenges our efforts to understand children in their richness and complexity (Sperry and Sperry; Wang et al.). These articles testify to the strengths of our methodological resources and creativity; nevertheless, several also challenge us to do better as they recognize the limitations of any one approach. The authors of these articles eloquently describe how the give and take of methodological practice can, on the one hand, interrogate unusual or controversial findings within a quantitative work through a more nuanced look at individual, cultural interpretations and belief practices (Huang and Kan); and can, on the other hand, provide the tools necessary to address the child's total linguistic competence in ways that detailed qualitative accounts may miss (Moreno et al.; Ward).

Despite the richness of these contributions, our task is not complete. We are struck by how few of these articles address language achievement past the early childhood years. One reason, we suspect, stems from the divides found in various siloed communities. As children grow, the focus shifts from understanding early language *acquisition* to explaining later differences in language *achievement*. However, language achievement is as multifarious as the speakers, communities, and languages involved. Although all parties in the conversation from scholars to policymakers to speakers themselves seek, in spirit, the best outcomes for children, the approaches taken by some parties often assume a one-size-fits-all, acultural solution that does not adequately address the diverse nature of on-the-ground reality.

For example, in recent years, much attention has been paid to early interventions to increase the amount of vocabulary heard in the homes of children living in poverty; this research has been driven by the goal of improving language achievement in the school years and assumes that later language achievement may be predicated upon a standardized approach to language acquisition. Yet, both the means of language acquisition and the outcomes of language achievement are embedded within rich cultural practices. Often, what language achievement means within the mainstream schooling context poorly translates to what it looks like in the broader contexts of families, communities, and even the marketplace. Once we focus on achievement from any single vantage point, we run the risk of measuring outcomes too narrowly and even of creating the possibility of gaming the system through artificial methods designed to remediate the identified “problem.”

This failure to determine what counts as achievement has generated one of the central tragedies in language studies over the past several generations, namely, the perpetuation of the myth of language deprivation within marginalized communities. Several of the articles in this Research Topic index the consequences of deficit thinking, whether it stems from political alliances, socioeconomic forces, or languages in contact. Wang et al. and Sperry and Sperry both suggest ways forward beyond this impasse, capitalizing on language strengths of all learners and acknowledging that language is as much a sociocultural practice situated within the various contexts of a child's life as it is an individual, cognitive, and social-emotional process set apart from meaning-making.

The focus on language strengths brings us back full circle to the reason we began this journey, namely, to query what children, families, and communities can tell us about language acquisition as it occurs on the ground, in their homes, and on an everyday basis. To the extent that any theory or intervention does not begin with this information, we suggest that it will remain ineffective and even harmful. We are reminded of Freire who insisted that theory makers, educators, and policymakers alike cannot operate in a vacuum. Our efforts must begin with the people we serve, remembering that the goal is not to “fix” people whose lives and practices are different from our own, but rather to understand our respective cultural contexts and ask how we can all move forward together.

## Author contributions

DS wrote the initial draft of the Editorial and is responsible for its final form. PS, EC, LS, and HS contributed suggestions and revisions to the manuscript.

## Conflict of interest

The authors declare that the research was conducted in the absence of any commercial or financial relationships that could be construed as a potential conflict of interest.

## Publisher's note

All claims expressed in this article are solely those of the authors and do not necessarily represent those of their affiliated organizations, or those of the publisher, the editors and the reviewers. Any product that may be evaluated in this article, or claim that may be made by its manufacturer, is not guaranteed or endorsed by the publisher.

